# Future Needs for Science-Driven Geospatial and Temporal Extravehicular Activity Planning and Execution

**DOI:** 10.1089/ast.2018.1838

**Published:** 2019-03-06

**Authors:** Jessica J. Marquez, Matthew J. Miller, Tamar Cohen, Ivonne Deliz, David S. Lees, Jimin Zheng, Yeon J. Lee, Bob Kanefsky, Johannes Norheim, Matthew Deans, Steven Hillenius

**Affiliations:** ^1^ NASA Ames Research Center, Moffett Field, California.; ^2^ Jacobs/NASA Johnson Space Center, Houston, Texas.; ^3^ SGT/NASA Ames Research Center, Moffett Field, California.; ^4^ ASRC Federal/NASA Ames Research Center, Moffett Field, California.; ^5^ Carnegie Mellon University, Silicon Valley/NASA Ames Research Center, Moffett Field, California.; ^6^ San Jose State University Research Foundation/NASA Ames Research Center, Moffett Field, California.; ^7^ Department of Aeronautics and Astronautics, Massachusetts Institute of Technology, Cambridge, Massachusetts.

**Keywords:** Planetary extravehicular activity, Planning and scheduling, Scientific data repository

## Abstract

Future human missions to Mars are expected to emphasize scientific exploration. While recent Mars rover missions have addressed a wide range of science objectives, human extravehicular activities (EVAs), including the Apollo missions, have had limited experience with science operations. Current EVAs are carefully choreographed and guided continuously from Earth with negligible delay in communications between crew and flight controllers. Future crews on Mars will be expected to achieve their science objectives while operating and coordinating with a science team back on Earth under communication latency and bandwidth restrictions. The BASALT (Biologic Analog Science Associated with Lava Terrains) research program conducted Mars analog science on Earth to understand the concept of operations and capabilities needed to support these new kinds of EVAs. A suite of software tools (Minerva) was used for planning and executing all BASALT EVAs, supporting text communication across communication latency, and managing the collection of operational and scientific EVA data. This paper describes the support capabilities provided by Minerva to cope with various geospatial and temporal constraints to support the planning and execution phases of the EVAs performed during the BASALT research program. The results of this work provide insights on software needs for future science-driven planetary EVAs.

## 1. Introduction

Mars extravehicular activities (EVAs), or planetary spacewalks, will face a host of new challenges when conducting simultaneous human and robotic operations on the surface while managing communication limitations with experts on Earth. As envisioned, human crew, together with robotic assets, will collectively be able to explore the planetary surface at a faster rate than any Mars rover mission to date (Mishkin *et al.,* 2007; Drake, 2009). However, the exact procedures and software tools necessary to conduct these spacewalks is unknown. Future EVA operations will integrate a vast amount of systems and operational knowledge to promote productivity and ensure mission success. This knowledge will be shared between the crew on Mars and the Earth-based engineering, operations, and scientific specialists who can only communicate with each other in a time-delayed communication environment (due to the distance between the planets). Additionally, future planetary EVAs will incorporate science-driven objectives, currently an integral part of deep space robotic missions (Hodges and Schmitt, 2011; Schmitt *et al.* 2011) but largely absent from all prior human EVA operations. As a result, there is a need to better understand how to support future planetary EVAs.

Present-day EVA operations are carefully choreographed and rehearsed events, planned to the minute by a large team of EVA engineers (Bell *et al.,* 2006; Bell and Coan, 2012; Miller *et al.,* 2015). Throughout execution, space-suited crew carefully complete tasks in microgravity while EVA flight controllers, located in the Mission Control Center (MCC), meticulously monitor life support telemetry and vehicle systems as well as track and manage task progress (Miller *et al.,* 2017c). The bulk of EVAs performed to date have focused on engineering objectives such as assembly, maintenance, and repair tasks (Portree and Treviño, 1997; Wilde *et al.,* 2002). Even Apollo lunar surface EVAs were heavily scripted, limited in mission duration and frequency, and performed with negligible communication latency with Earth-based support personnel (Miller *et al.,* 2016b, 2017a).

Future planetary EVAs will have to distill broad scientific research objectives into operationally useful constructs necessary to support successful science-driven EVA operations. To this end, the BASALT (Biologic Analog Science Associated with Lava Terrains) research program aimed to study and understand the processes and the underlying software capabilities necessary for planning and executing science-driven EVAs on Mars. Within BASALT, a suite of software tools, collectively known as Minerva, was used as the primary software platform that provided integrated support for EVA planning and execution. Minerva is composed of three software tools: Exploration Ground Data Systems (xGDS), Playbook, and Surface Exploration Traverse Analysis and Navigation Tool (SEXTANT), subsequently described.

## 2. Background: Supporting EVA Operations

This paper describes the necessary future software capabilities to support two key phases of planetary EVA operations: planning and execution. It builds upon previous research investigating analogous planetary EVA planning and execution (*e.g.,* Marquez and Newman, 2007; Chappell *et al.,* 2017; Miller *et al.,* 2017c). Embedded within both phases of operation is the need to cope with the geospatial and temporal aspects of EVA. Geospatial planning involves coordinating crew and assets to maneuver to and execute desired tasks within specific regions or at targets of interest using *a priori* generated maps (*e.g.,* from satellites) and route planning (or path planning) (Cummings *et al.,* 2012; Roth, 2013). Temporal planning refers to the sequencing of events through which tasks and objectives are executed by crew who are following the scheduled timeline and task procedures (Bell *et al.,* 2006; Miller *et al.,* 2017d). Subsequently, we describe the support capabilities provided by Minerva to cope with various geospatial and temporal constraints to support the planning and execution phases of the EVAs performed during the BASALT research program.

### 2.1. EVA planning

**FIG. 1.**
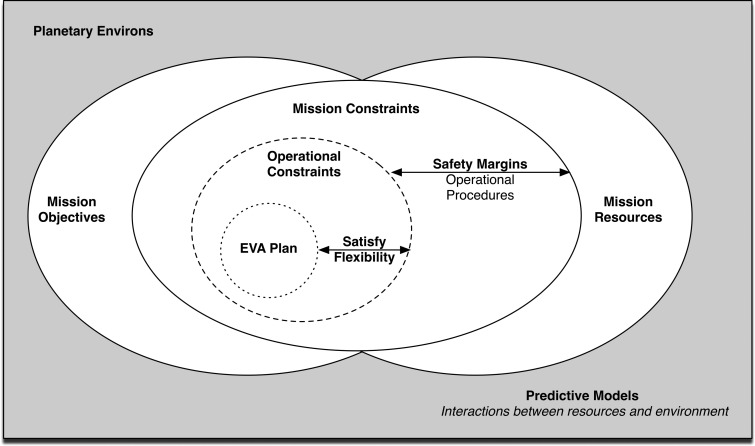
EVA planning needs overview, adapted from Marquez and Newman (2007).

Extravehicular activities must first be planned before they can be executed. An overview of the required information to plan future planetary EVAs is illustrated in Fig. 1 (Marquez and Newman, 2007). First, a set of EVA mission objectives must be defined to meet science and engineering objectives, which in turn are constrained by the available mission resources. On Mars, mission resources include transportation options and the amount of consumables (*e.g.,* oxygen) available for the EVA. Mission constraints and safety margins are operationally predefined before a plan can be created; for example, a safety margin may be a time of day by which the EVA must be finished (*i.e.,* a hard stop). Finally, an EVA plan is delineated, which may include routes, schedules, and specific tasks for astronauts to complete.

For BASALT, the scientific objectives were defined by the interdisciplinary Science Support Team (SST) (discussed more extensively in Brady *et al.*, 2019). The acquisition of scientific knowledge through observation, measurement, and collection of physical samples was the underlying motivation and influential factor that shaped all EVA operations. The mission resources available to conduct the EVA included the mission personnel, the available instruments, and the communication infrastructure. BASALT also followed a set of EVA Flight Rules that imposed safety margins on EVA execution and constraints on how an EVA could be designed (Table 3, Beaton *et al.,* 2019). For a BASALT EVA, defining a plan required identifying the specific science objectives, selecting areas or locations where appropriate samples were to be obtained, and laying out the route to traverse to reach these locations. The EVA plan was scheduled such that all the relevant scientific data could be acquired while giving the scientific experts, located in a time-delayed Earth-based Mission Support Center (MSC), sufficient time to provide guidance. This affordance with each EVA was enabled by visiting scientific worksites repeatedly whereby each sampling priority could be refined and decided upon through more targeted information gathering (see Beaton *et al.*, 2019, for EVA timeline details).

One planning aspect that is not illustrated in Fig. 1 is the difference between strategic and tactical EVA planning. For the purposes of this paper, strategic planning refers to any EVA planning that occurred before the start of a BASALT mission deployment. This phase of planning is characterized by the science team discussing scientific priorities and identifying ways to meet those objectives (see also Brady *et al.,* 2019). Tactical planning refers to creating both geospatial and temporal plans for an EVA (discussed in this paper). Tactical decision-making is covered separately (see Stevens *et al.,* 2019).

### 2.2. EVA execution

In order to execute, or complete, the campaign of planned EVAs, there are key functions that must be supported (Table 1). These work functions are what currently characterize EVA execution and are managed principally by Earth-based EVA flight controllers. For future planetary EVAs, the distribution of these work functions across Earth-based EVA flight controllers, extravehicular (EV) astronauts, and intravehicular (IV) astronauts is still being investigated. Arguably, at least one IV crew counterpart colocated with the EV crew will need to manage some of the work functions since EVA flight controllers will have to contend with communication latency and limited bandwidth restrictions (Abercromby *et al.,* 2013a, 2013b; Miller *et al.,* 2017c).

**Table 1.** Support Capabilities for EVA Execution, Miller *et al.* (2017b)

*Work function*	*Description*
Timeline management	Tracking progress on tasks, time, and location.
Life support system management	Tracking consumption of life support systems (*e.g.,* oxygen, power, water).
Physiological management	Tracking crew physiological state (*e.g.,* exertion, heart rate).
Communication management	Overseeing multiple sources of communication, including video, audio, and text interchanges.
Science operations management	Real-time processing of science data and products, including categorization.

For the BASALT research program, we follow the concept of operations where two IVs and members of the MSC and SST manage the work functions identified in Table 1. The SST could electronically chat with IVs while reviewing the incoming science data. The IVs served as an “information relay” between the EVs and SST, buffering, filtering, and assimilating information in a way that was easier for the EV crew to consume, much as an Earth-based flight controller does for a crew in near-Earth space. IVs directed EVs to planned locations and kept them on schedule. The BASALT research program concepts of operations as well as EV, IV, and MSC roles and task assignments are further outlined in Beaton *et al.* (2019).

Most of these work functions were simulated in the EVAs with the exception of life support system management, which was not a focus of BASALT (see Lim *et al.,* 2019). Additionally, the 4-hour EVAs in BASALT only included the relevant scientific tasks prioritized for the day. The EVAs included science exploration and sample collection but did not include returning back to home or habitat (see Beaton *et al.,* 2019). Hence, the primary motivation for the BASALT EVAs was to achieve science objectives instead of prioritizing life support and physiological constraints (and to a lesser extent, timeline constraints).

For the BASALT research program, Minerva provided the software capabilities for planning and executing planetary EVAs that supported specifically timeline management, communication management (with the exception of voice communications), and science operations management. By using Minerva in an analogous planetary EVA, we were able to observe and evaluate which capabilities were essential and identify missing capabilities needed to successfully support future human exploration of Mars.

### 2.3. Minerva

Minerva is composed of three software tools that support one or more of the key work functions required for planning and executing science-driven planetary EVAs (Table 2): xGDS, Playbook, and SEXTANT (Deans *et al.,* 2017). xGDS and Playbook have been deployed in a variety of analog field tests such as Desert Research and Technology Studies (Desert RATS), NASA Extreme Environment Mission Operations (NEEMO), and Pavilion Lake Research Program (PLRP) (Lim at al., 2011; Deans *et al.,* 2013; Heldmann *et al.,* 2015; Marquez *et al.,* 2017). Through these field tests, our team focused on developing effective user experiences for each software tool. While each respective tool had been developed separately prior to BASALT, they were all designed to be easy to use with limited training. As xGDS and Playbook have been incrementally and independently matured to support test and science objectives for different field tests, BASALT is the first analog field test where both tools have a set of common objectives to support analog EVA operations.

**Table 2.** Mapping of Minerva Software and EVA Work Functions

	*Minerva*		
*EVA work function*	*xGDS*	*SEXTANT*	*Playbook*
Timeline management	P&E	P	P&E
Science operations management	P&E		
Communication management	P&E		E

E = Execution only; P&E = Planning and Execution; P = Planning only.

**FIG. 2.**
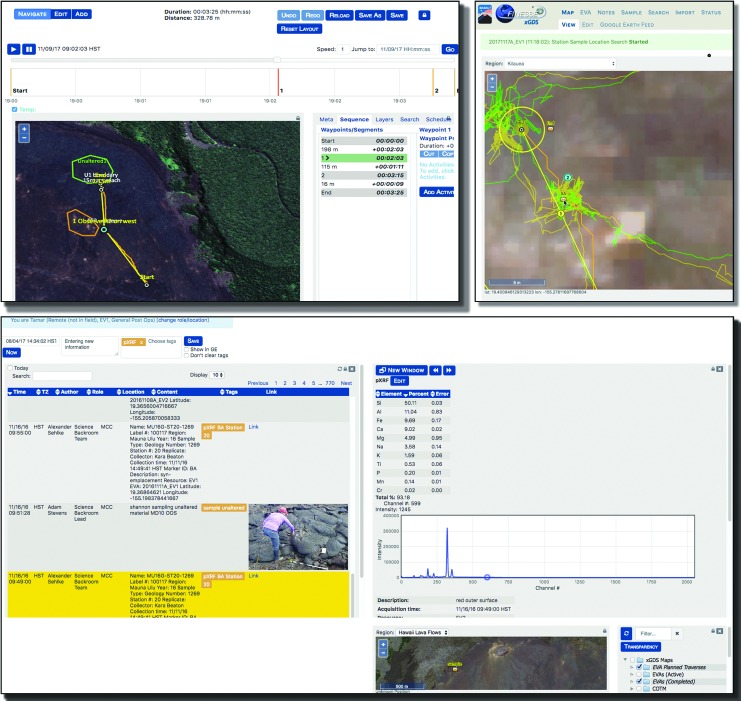
Overview of xGDS software, including traverse planning (top left), tracking EVA (top right), and time-stamped, geolocated notes, photos, and instrument data (bottom).

xGDS supports planning, monitoring, data archiving, and data exploration (Lim *et al.,* 2011; Deans *et al.,* 2013; Lee *et al.,* 2013). Using xGDS, the team can use *a priori* generated maps to create and share geospatial information in order to develop scientific traverse plans (Fig. 2). Additionally, xGDS serves as a digital repository for operational and scientific data, tracking and monitoring of EV positions, video, photos, sample metadata, and science instrument data. This data can be annotated via integrated, geolocated, and time-stamped digital notes. All collected data is stored in searchable databases for real-time or post-mission search and analysis.

SEXTANT is a resource-based path planning tool which optimizes human traverses based on a variety of cost functions, specifically distance, time, or energetics (Johnson *et al.,* 2010; Gilkey *et al.,* 2011). Once the traverse plan was created in xGDS, SEXTANT calculated a more granular path between waypoints, only taking the terrain elevation into account. SEXTANT modeled the energy a human would expend crossing a given terrain and suggested an optimal route that would take the least energy, time, or distance based on user input. The resulting path, timing, and distance estimates from SEXTANT were returned to and displayed within xGDS (Norheim *et al.,* 2018).

**FIG. 3.**
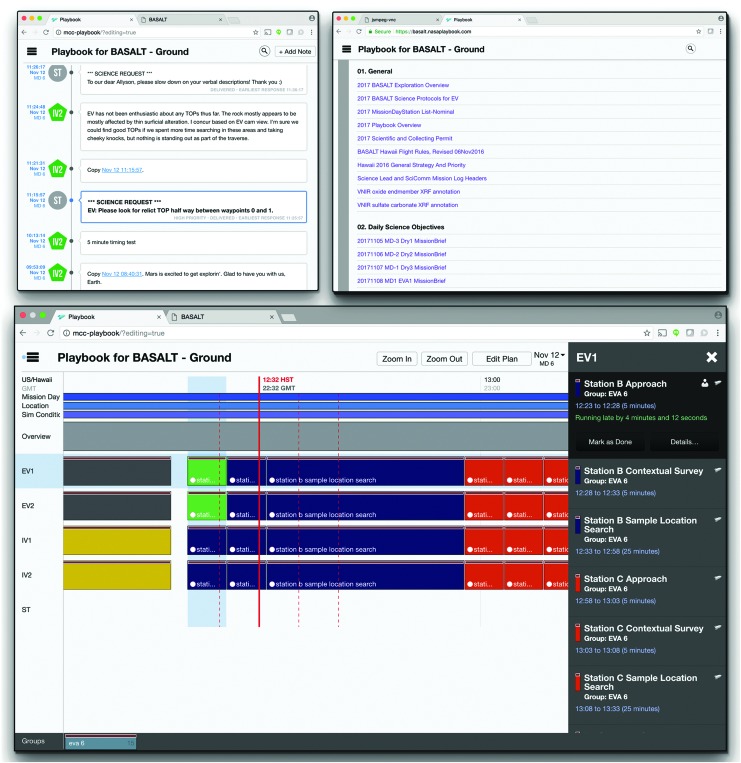
Playbook views, including Timeline (bottom), Mission Log (top left), and Procedures (top right).

Playbook is a mobile, timeline execution tool aimed at assisting astronauts with following and completing assigned activities (Marquez *et al.,* 2013). The Timeline (*i.e.,* a visualization of scheduled activities on a given day) and corresponding constraints are planned, viewed, manipulated, and statused in Playbook^1^. Alongside the schedule, Playbook provides the Mission Log, a multimedia chat interface. Crew and ground teams can share information and coordinate tasks by leveraging both the Mission Log and the real-time crew activity status of the Timeline. Playbook also includes a simple centralized procedure/document repository (Fig. 3).

### 2.4. Minerva users

There were two user groups for Minerva: the crew on an analogous “Mars” and the Mission Support Center (MSC) on analogous “Earth” which includes the Science Support Team (SST). The SST was a large team of diverse scientists, with varying backgrounds and expertise to meet the BASALT research program's science goals (Lim *et al.,* 2019). Crew was a mixture of scientists and engineers, two in the “Mars” habitat (IVs) while the others (EVs) physically explored the terrain (Lim *et al.,* 2019, Fig. 2). IV1 and EV1 focused on ops tasks while IV2 and EV2 emphasized science tasks (Beaton *et al.,* 2019). While all users had access to the Minerva software tools, some roles did not require the use of certain tools, and some users did not have access to them due to hardware limitations.

The mission personnel on “Earth” were responsible for EVA planning, and they used all the Minerva software to support timeline, science operations, and communication management (see Tables 2 and 3). The SST used xGDS for the majority of the EVA planning, including construction of traverse plans and reviewing previously collected science data. A significant portion of their time using xGDS was spent during the predeployment, strategic planning phases, identifying regions of interest and studying the various science maps available to them through Minerva (see also Brady *et al.,* 2019). Tactical planning occurred during the deployment, when the SST finalized traverses to specific locations that met their science objectives. SEXTANT was used to calculate optimized human traverse paths for each EVA timeline. The EVA Planner, a personnel role in the MSC, scheduled all the team's activities in Playbook. During EVA execution (Table 4), the MSC relied on xGDS to track crew progress along their planned traverse routes and Playbook to track completion of activities. They also used the Playbook Mission Log for text communication and image sharing between “Earth” and “Mars.” The SST utilized xGDS for reviewing and annotating all the science data being received from “Mars,” including video, photos, and instrument data.

**Table 3.** Generalized Functions of Minerva Software Components Utilized in Planning EVA Phase

*EVA work function: Planning*	*xGDS*	*SEXTANT*	*Playbook*
Timeline management	Planning traverses	Optimizing traverse paths between stations	Scheduling activities
Science operations management	Reviewing previously collected science data	—	—
Communication management	Reviewing previously collected video	—	—

**Table 4.** Generalized Functions of Minerva Software Components Utilized in Execution of EVA Phase

*EVA work function: Execution*	*xGDS*	*SEXTANT*	*Playbook*
Timeline management	Tracking progress along traverse	—	Tracking completion of activities
Science operations management	Reviewing and annotating incoming science data	—	—
Communication management	Viewing and annotating incoming video	—	Sending and receiving text and image communications

Audio communication is managed outside of Minerva.

The EV and the IV crew members used Minerva to execute EVAs, although their interactions with Minerva were different due to the fact that EVs had limited display hardware. EVs had a wrist display (an iPhone 6) that let them view^2^ traverse plans created in xGDS, alongside their current position, tracks, and important locations on the map identified by the IVs. Occasionally, EVs would view text or images shared through Playbook on the wrist display. Similar to the MSC, the IVs also leveraged xGDS to track EV progress and review and annotate science data. IVs used Playbook for text communication and image sharing with the SST. (For the 2016 BASALT deployments, IVs did not use the timeline view to track activity progress.)

### 2.5. Information flow within Minerva

As described in the works of Lim *et al.* (2019) and Beaton *et al.* (2019), EVs conducted the EVA, guided by IVs, while the SST and MSC followed along and provided recommendations during the EVA execution. The general flow of an EVA was as follows: EVs explored areas that were of interest to the science team, describing the location and taking pictures; EVs with IVs' guidance proposed candidate locations for possible scientific measurements and sample collection; the SST provided feedback on the desirability of proposed locations; and EVs collected the scientific samples.

As the EVA was executed, generated data was shared through Minerva. The data in turn supported the information needs required for the IVs, SST, and MSC to maintain a minimum level of situation awareness, which was inferred but not measured. Situation awareness (SA) is defined as “the perception of the elements in the environment within a volume of time and space, the comprehension of their meaning and the projection of their status in the near future” (p. 36, Endsley, 1995a; Endsley, 2000). Applying this definition to BASALT EVAs, situation awareness meant the IVs, SST, and MSC were able to (1) perceive (see) the data that the EVs communicated (via voice, photos, and video); (2) comprehend or understand the meaning of the information in the context of the EVA (*e.g.,* EV is at third sample candidate locations); and (3) project the future state of the EVA (*e.g.,* when EVs might be done with a particular EVA phase). Additionally, data and information are distinct from each other (see also Endsley, 1995b); data are the bits and files exchanged through Minerva, while information is a comprehended intent. For example, a text message is data while science priorities contained in that text are information. This distinction is relevant as recommendations for future needs are framed in the context of information and functionality.

**FIG. 4.**
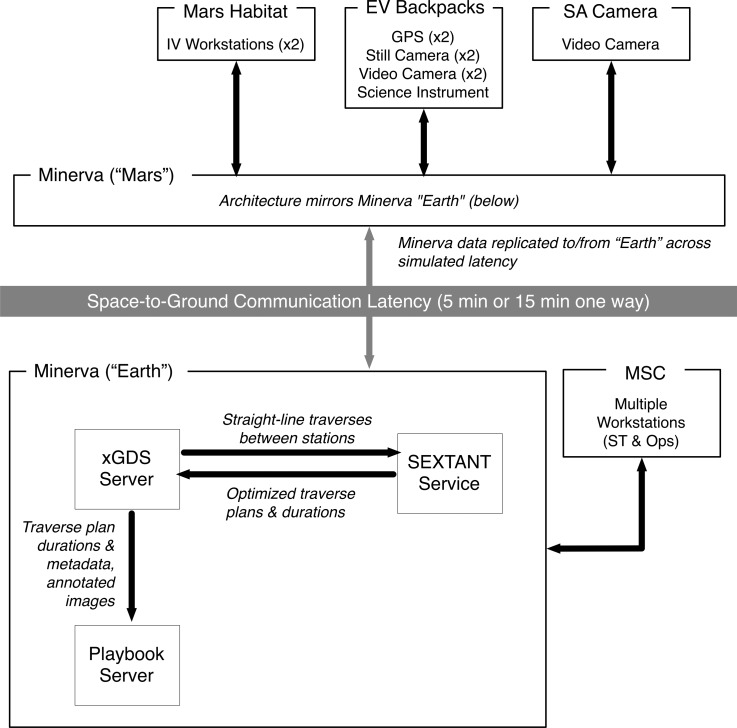
High-level Minerva architecture, showing data flow between hardware and software components (xGDS, Playbook, and SEXTANT) across simulated communication latency.

The BASALT mission personnel were able to perform the various EVA work functions because Minerva enabled them through the bidirectional data flow between “Mars” and “Earth” during EVA execution (Fig. 4). The data provided the means by which information required by the various users was exchanged to perform their assigned roles and responsibilities. Table 5 lists the data pertinent to a BASALT EVA that flowed through Minerva. The data was exchanged while operating under simulated Earth-Mars communication conditions (the one-way latency times and bandwidth restrictions were predetermined by the BASALT research program, Lim *et al.*, 2019, and Beaton *et al.*, 2019). The simulated communication latency was implemented by deploying two matching sets of Minerva hardware and software services (Playbook, xGDS, and SEXTANT). Data generated on or saved to the “Mars” servers (Minerva “Mars”) was replicated to the “Earth” Minerva server (Minerva “Earth”) after the designated communication latency, that is, after 5 or 15 minutes. Data generated on “Earth” followed the identical path in the opposite direction. During low-bandwidth simulation conditions, the video data was suppressed on “Earth.”

**Table 5.** Data Flow between EVs, IVs, and SST through Minerva

*Data*	*Data type*	*Created by*	*Accessed by*	*Special attributes*	*Latency provided by*
Planned	Planned traverse	SST	SST, IV, EV	EV views on wrist display	xGDS
	Planned EVA activities	MSC	SST, IV	EV can access if desired	Playbook
Execution	Telemetry, position	EV	SST, IV	Tracks also visible. All geospatial data also visible in maps.	xGDS
	Notes and tags	IV, SST	SST, IV	Time-stamped and geolocated	xGDS
	Instrument data	EV	SST, IV	Instrument data automatically transmitted and geolocated (only pXRF)	xGDS
	Sample metadata	IV	SST, IV	Time-stamped and geolocated	xGDS
	EVA activity statuses	EVA Planner	SST	IV can access if desired, other aid	Playbook
	Photos	EV	SST, IV	Time-stamped and geolocated	xGDS
	Text/image communication	IV, SST	SST, IV, EV	EV can access if desired	Playbook
	Video	EV	IV, SST	Audio track on video. SST sees delayed video.	xGDS
	Voice communication	SST, IV, EV	SST, IV, EV	Recorded and played under delay	*Outside of Minerva*

Most of the “Mars” data (*e.g.,* position telemetry, photos) were generated in the field by EV1 and EV2 as they explored the volcanic terrain during an EVA. This data was generated and transmitted through a backpack that each EV wore, which is described and visualized in the work of Lim *et al.* (2019). The following is a brief overview of the hardware network architecture that enabled data flow during the BASALT deployments.

The backpack was equipped with wireless meshing networking gear,^3^ GPS tracker, chest-mounted video camera, and handheld still camera with a wireless link to the backpack. Additionally, EV2 utilized a suite of portable handheld scientific instruments that transmitted data wirelessly to the backpack. All data generated by EV1 and EV2 were transmitted to the Minerva “Mars” servers via a meshing wireless network. There was a third video camera (SA camera) which also transmitted its feed through the wireless network. IVs had instant access to the EV-generated “Mars” data once stored in Minerva. There was negligible communication latency between EVs and IVs since the IVs were located on the “Mars Habitat.” Other “Mars” data (*e.g.,* text messages, science notes) were created by IV1 and IV2 when they used Minerva “Mars.”

Only the science and mission support team members used the Minerva “Earth” servers in order to access, monitor, and generate data. The SST and MSC were physically colocated and often shared the same projected views of xGDS and Playbook. All of the “Mars” data they saw or heard was delayed according to the simulated conditions; likewise, any data generated from the SST arrived to the IVs after the communication delay. Figure 4 shows the interconnections and data flow between the various Minerva software components, including the data transfer between the “Earth” and “Mars” sides of the communication latency. See the work of Cohen *et al.* (2019) for further technical details of Minerva software integration. Payler *et al.* (2018) describe the MSC setup, including where Minerva interfaces were displayed. Deans *et al.* (2017) describe MSC, IV, and EV interface access.

**FIG. 5.**
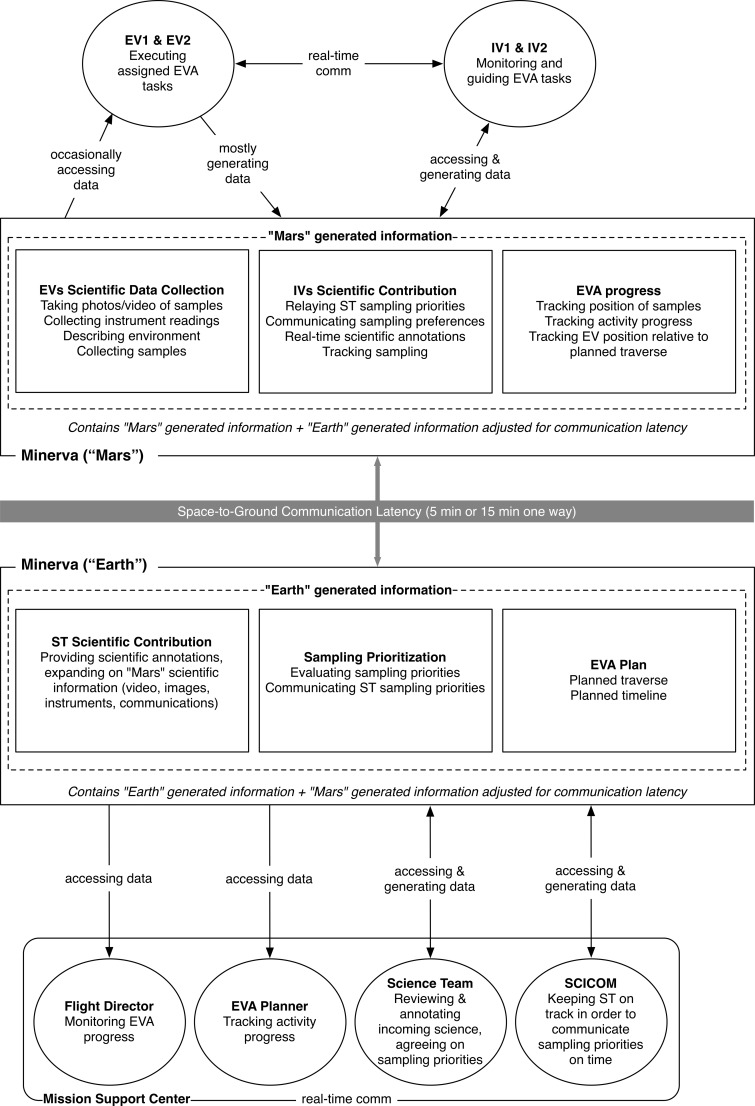
Information flow across Minerva and users' tasks during EVA execution.

While Fig. 4 shows how the data flows through the Minerva architecture, Fig. 5 illustrates the information exchanged during EVA execution between Minerva users. For instance, in order for the SST to provide feedback on which candidate location was a suitable site to collect scientific samples, they must first assess all the candidate locations. They reviewed the video, images, and instrument data collected on the candidate location and correspondingly wrote and shared scientific observations related to the location. In the meantime, IVs tracked how EVs were progressing through the timeline, ensuring that the SST had sufficient information and time to provide feedback on which samples to collect. Subsequently, the SST sent text messages to the IVs, indicating their sampling location preferences. All of these interactions flowed through the Minerva tools during EVA execution.

## 3. Supporting the Planning of a Planetary EVA

### 3.1. Geospatial planning: Identifying exploration sites and routes

**FIG. 6.**
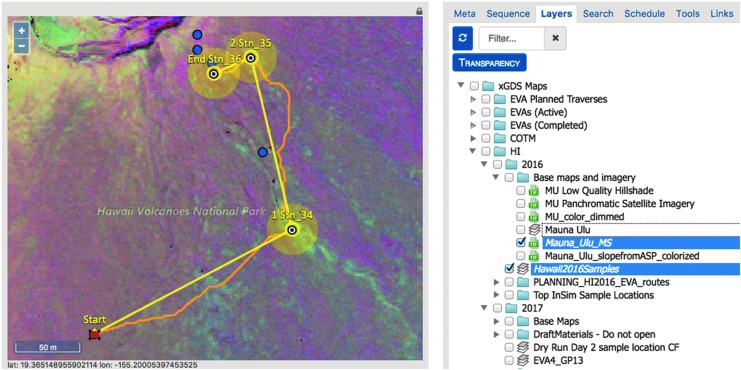
Planned traverse and map layers in xGDS Traverse Planner.

Each EVA is composed of both a geospatial plan and a temporal plan designed to address the scientific goals and objectives of the day. The geospatial plan identified areas of scientific interest and defined a traverse path to reach them. The process of developing a geospatial plan first involved organizing map data in xGDS to focus the planning problem. Map data included terrain ortho-imagery, elevation and spectral data (Table 6), and previously identified regions of interest (*i.e.,* map markup, imported from Google Earth or created directly within xGDS). Other geolocated data acquired from previous EVAs or fieldwork activities could be searched and rendered on maps. As listed in Table 5, this data included notes, photos, sample locations, and instrument data. Prior EVA traverse plans and actual traverse tracks could also be overlaid (see Brady *et al.*, 2019, for more details). Once this information was collated, members of the science team used xGDS to create detailed map-based traverse plans for the EVAs (Fig. 6). Providing a centralized map repository for organizing and viewing maps and markups facilitated the science planning process.

**Table 6.** Map Data Available in xGDS for BASALT Deployment

*Source*	*Description*
UAV	Orthophoto mosaics (2–5 cm/pixel)
Satellite	Pan-sharpened true color basemap (∼1 m/pixel)
	Mineralogical parameter map (∼1 m/pixel)
UAV orthophoto	Hillshade
	Colorized elevation
	Colorized slope
Aerial LIDAR	Hillshade (1 m/pixel)
	Colorized elevation (1 m/pixel)
	Colorized slope (1 m/pixel)

During traverse planning, the scientists, using a click and drag interface, could quickly map out a series of stations (waypoints on the map) connected by straight lines. xGDS provided a high-level summary of distances and approximate durations to traverse these distances based on a constant estimated speed. The scientists could control a variety of metadata regarding components of the traverse such as labels, size of the station boundary, descriptions, and activities to be done at each station.

**FIG. 7.**
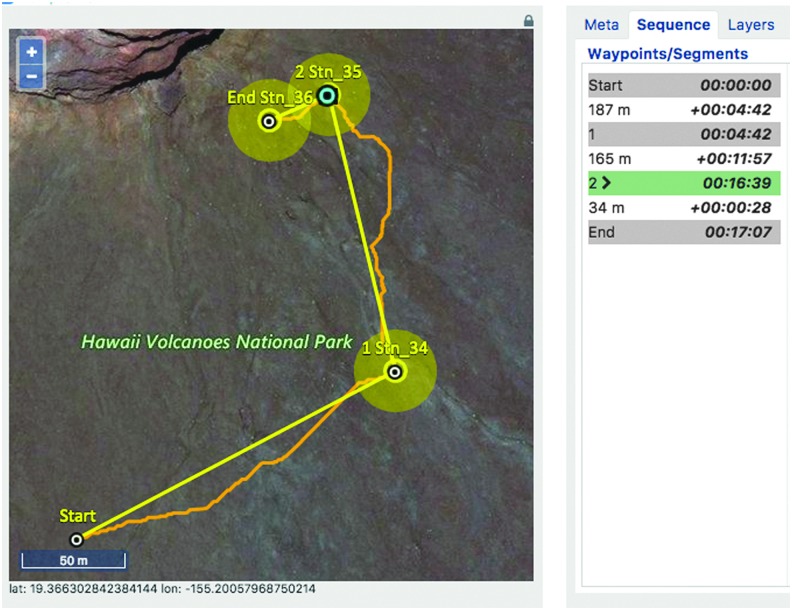
xGDS showing stations (yellow circles), SEXTANT (curved orange lines between stations), and SEXTANT timing (right panel).

Once a traverse plan was finalized (Fig. 7), it could be scheduled in xGDS. This made the traverse plan accessible to the IV and EV crew. The completed traverse plan could be viewed from xGDS, from Google Earth, or from a map view the EV crew could see on their wrist-mounted displays.

### 3.2. Temporal planning: Scheduling a set of EVA activities

The generation of EVA timelines for BASALT involved a number of iterative steps. A timeline was composed of a sequence of activities each with a planned duration and assignment to the relevant flight personnel (*e.g.,* EV, IV, SST) that fit the predefined maximum duration of 4 hours. These activities were defined initially in a spreadsheet. Per the EVA plan, each discrete activity for the EV crew was assigned to both crew members to mimic a “buddy-system” style of EVA operations (*i.e.,* both EVA crew members always performed the same activities). A representative set of timelined activities can be found in the work of Beaton *et al.* (2019, Table 2).

To view the EVA timeline in Playbook, the EVA Planner first created the temporal plan in Analog Score^4^. The temporal plan consisted of a set of EVA activities, durations, assignments, and estimated start times. Once EVA activities were scheduled, a few supporting activities were included to provide temporal relations during execution. Given the aim of BASALT to better understand how well a SST could actively engage and influence EV/IV operations, the timeline included SST “bingo times” in temporal relation to relevant EVA activities. The SST “bingo time” signified the latest time the SST could provide science decisions to IV before execution of the next scheduled activity. The “bingo time” took into account the simulated communication latency to ensure information arrived at the appropriate times. These activities allowed the SST to quickly see if they were still able to provide feedback to the EV/IV regarding upcoming operations in a timely fashion.

**FIG. 8.**
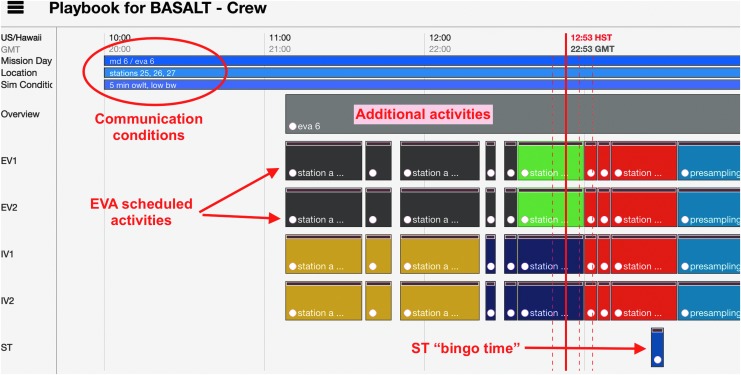
Annotated Playbook timeline view.

Additional activities in the timeline included overview activities, “hard stops,” and EVA margin derived from flight rules. Overview activities had links to documents^5^ related to the day's science overview and objectives. “Hard stops” denote periods in the timeline where activities must conclude per Flight Rules. Due to sunlight constraints, EVAs could operate up to 16:00 local time but no later. EVA margin (as defined per Flight Rule EM7, Table 3 in Beaton *et al.*, 2019) stipulated that an EVA may extend operations up to 30 minutes beyond nominal operations in order to complete activities. These activities were scheduled to aid the enforcement of the flight rules during execution and visualize timeline constraints imposed on EVA execution. Finally, the Playbook timeline also showed other relevant information about the EVA. During the 2016 deployment, the simulated communication latency and the names of station locations to be explored were visible in the timeline view as shown in Fig. 8.

Based on previous analog research, the EVA timeline design had sufficient ground assimilation time (GAT) as a function of communication latency, that is, provide enough time for the SST to see and review scientific EVA data and to give their recommendations. Sufficient GAT was based on previous analog research and was at least 1 hour and 45 minutes to 3 hours and 10 minutes, depending on the EVA (Chappell *et al.,* 2016; Miller *et al.,* 2016a; Beaton *et al.,* 2017). Ideally, the EVA temporal plan would closely and accurately estimate the duration of each activity completed by the EV, including approximations for the geospatial plan. The content of the EVA activities, their sequencing, and their durations were initially established based on previous research and refined based on engineering tests before the deployments (Beaton *et al.,* 2019). Unfortunately, traverse duration estimates calculated by xGDS and SEXTANT were not sufficiently accurate and, hence, not reflected in Playbook.

### 3.3. Planning process limitations

While the science team could create traverses in xGDS, they could not create fully detailed EVA timelines in that tool. As a result, there were planning process limitations that Minerva could not support. First, xGDS could only associate activities to waypoints, and the EVAs were designed to do activities along the traverse and at waypoints (or stations). Second, EVA activities were represented in multiple tools because xGDS, Playbook, and spreadsheets to track EVA timelines were not integrated nor built to be synchronized. Finally, based on the EVA design, the location of activities was not predetermined. For instance, there could be two stations to explore in one EVA, but it was not known before execution which station would be selected by the SST for sampling (as this was determined during execution).

## 4. Supporting the Execution of a Planetary EVA

### 4.1. Tracking EVA progress

Once an EVA started, Minerva supported tracking EVA execution along two main dimensions: geospatial and temporal. In general, geospatial tracking meant that Minerva users were able see where EVs were located during the EVA execution and where data was generated or collected. All traverse tracking was facilitated via xGDS. The IV and MSC watched position and track information via xGDS in Google Earth superimposed over the planned traverse. EVs viewed their planned traverse, their current position, and past tracks on a map through Google Earth on their wrist-mounted display. While the EVs could see this information and could follow the planned traverse, they mostly received verbal directions and guidance from the IVs who were also tracking the same information. Additionally, all the data that went through xGDS was both time-stamped and geolocated. This capability allowed IVs to quickly create geolocated notes that could be viewed on the xGDS maps (and seen by EV and, subsequently, SST and MSC). Images taken by the EVs were also geolocated and could be found on xGDS maps. All the SST notes created in xGDS were correctly geolocated (*i.e.,* appropriately synced based on the simulated latency between the two Minerva servers). Occasionally, the SST marked a particular EV position through xGDS for scientific reasons; IVs subsequently received this geo-marked information after the communication latency passed.

In terms of temporal tracking, all data generated or collected was time-stamped and EVA activity progress was shared among the MSC. During an EVA, the EVA Planner (or an out-of-sim person on the “Mars” side) updated the scheduled activities in Playbook to the as-run time and statused them as started, completed, or aborted. The SST and MSC were able to track EVA activity progress in this manner. The SST viewed the updates on Playbook's timeline and adjusted their decision-making process to the new estimates. As previously mentioned, for 2016 BASALT deployments, IVs tracked EVA activity execution through a spreadsheet (which was not integrated into Minerva).

**FIG. 9.**
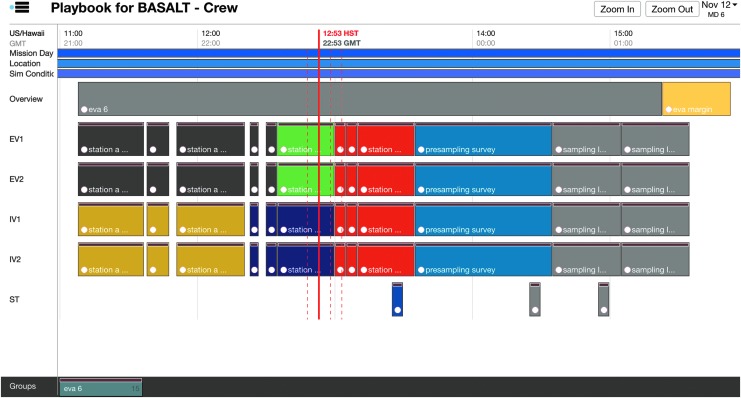
Playbook Timeline, showing multiple EVA activities and assignments. Solid red line is current time, and dashed red lines are aids to estimate communication latency.

Throughout execution, Playbook showed the current time over the timeline with a solid red line, known as a Marcus Bains Line. This is similar to timeline viewing in real-time International Space Station operations^6^. While conducting EVA operations under communication latency, Playbook provided three additional red dashed lines (defined here as Marquez Bains Lines) which were visual indicators of the communication latency between “Mars” and “Earth” to further assist in temporal tracking of EVA. As shown in Fig. 9, the leftmost dashed line is one-way time-delay in the past; the next dashed line is one-way time-delay in the future, while the rightmost dashed line is the round-trip time in the future. This is best described through an example. If the SST received a message, the leftmost dashed line would indicate when it was sent by the IV. If the SST sent a message, the IV would receive that message at the time indicated by the next dashed line. The SST would expect the earliest possible time to receive a reply from the IV would be the time indicated by the rightmost dashed line. These visual indicators were intended to help Playbook users to take into account the communication latency between “Mars” and “Earth,” particularly when trying to understand the delayed data being received as well as deciding whether upcoming communication could impact future tasks.

While not technically geospatial or temporal tracking, it was observed both IV and the MSC tracked EVA execution by closely monitoring the multiple video feeds available to them in relation to the synchronized EV crew position and tracks. Video streaming from EV chest cameras and the SA camera provided context of the surrounding environment as well as immediate feedback as to what was going on in EVA. xGDS recorded and supported viewing of multiple video streams alongside EVA tracks both in real-time and after the communication latency. The SST and MSC received the video after the communication latency.

### 4.2. Enabling decision-making across teams

It is essential for the science team not only to track EVA execution but also to maintain sufficient situation awareness of the large inflow of scientific information coming from EVs and IVs in order to then provide guidance on the scientific samples they would prefer. Thus, a key element to consider is enabling the science team to make scientific decisions that can be delivered in time to influence how the EVs execute the EVA. For a detailed discussion of the scientific rationale and processes involved in the tactical decisions being made, see the work of Stevens *et al.* (2019). The remainder of this section discusses the underlying software capabilities that enabled the exchange of information and priorities among the BASALT team. Minerva provided the underlying database infrastructure and user interfaces to coordinate and integrate the various video, photos, notes, and instrument data (under communication latency) to support EVA execution and time-sensitive scientific decision-making.

**FIG. 10.**
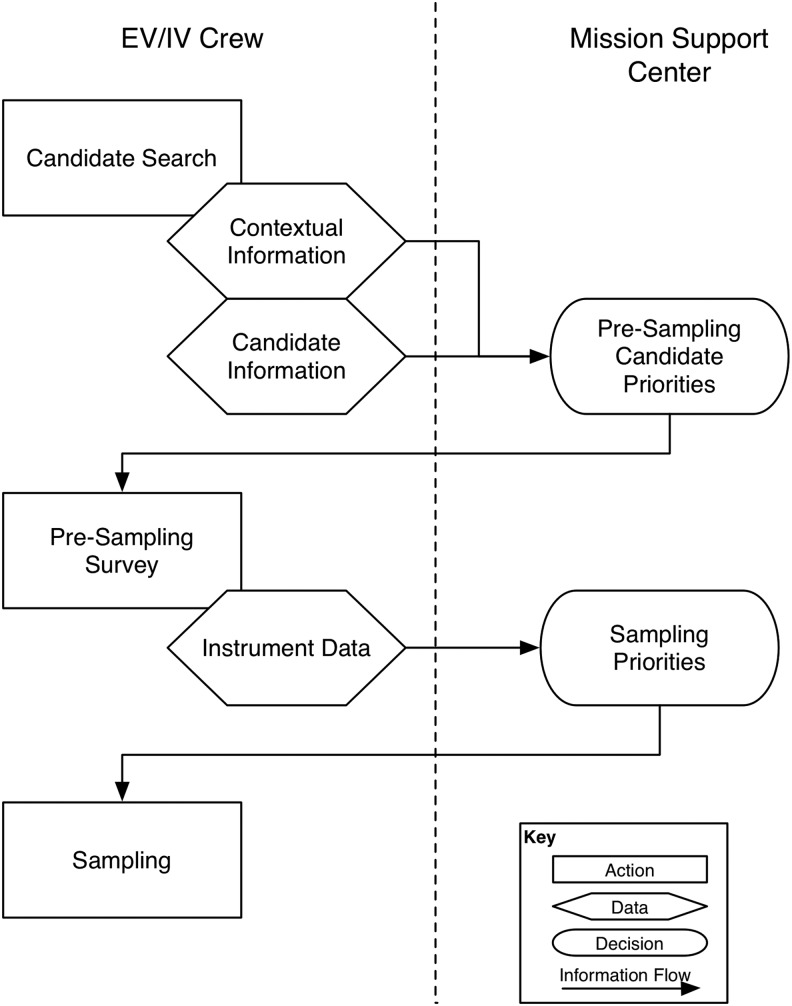
Science team priorities and critical decisions during EVA execution.

Based on the BASALT EVA timeline structure (see Beaton *et al.,* 2019, Table 2), two key decision points were scheduled for the science team to convey priorities to the IVs: (1) where to conduct presampling survey and (2) where to sample (Fig. 10). Generating these priorities involved considering both the tactical and strategic science objectives while synthesizing the data provided by the EV/IV crew throughout execution (Brady *et al.,* 2019; Stevens *et al.,* 2019). To assist in this process, Mission Briefs were generated to articulate the scientific priorities associated with each individual EVA, balanced across the competing scientific aims of the various scientists (Brady *et al.,* 2019). The Mission Briefs were available to the entire MSC through Playbook.

**FIG. 11.**
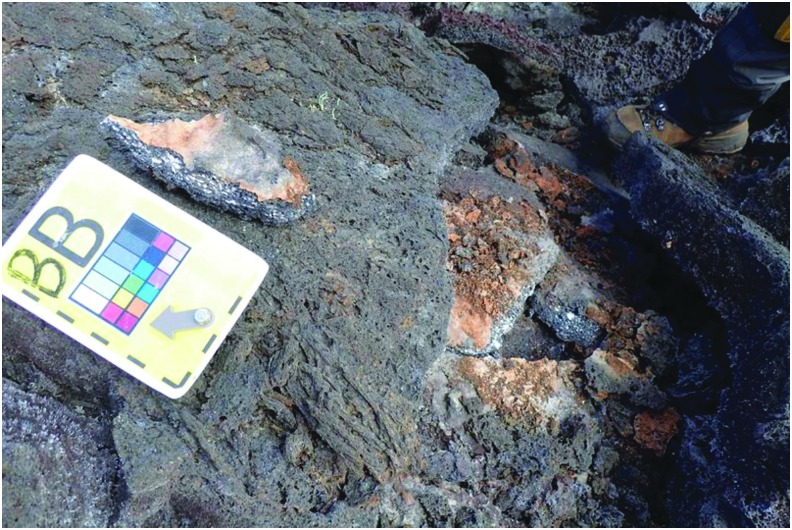
Example image from EV identifying a potential candidate sample with lettered marker.

In order to make the presampling decision, the SST had to see and hear the scientific information gathered by the EVs and IVs during the Candidate Search. The scientific information centered around providing contextual and candidate sample information, everything from overall environment to porosity of candidate sample. This information was provided through several data streams. The SST saw the video from a chest-mounted camera on each EV and from the SA camera. The chest-mounted cameras approximately provided the EV's point of view while the SA camera was aimed to show the context of both EVs working. In addition, the EVs captured photos with handheld cameras to provide additional detailed context of the surrounding terrain and area being explored. When the EVs entered the predesignated stations to be explored, they dropped lettered markers on potential sample locations, followed by close-up photos of these locations (Fig. 11). The EV/IV dialog throughout the presample survey phase of the timeline provided a first-person account of the terrain being examined which played a valuable role in the SST decision-making process.

During this EVA phase, the SST had to manage, review, and discuss the various scientific information streaming through Minerva. As mentioned, Minerva provided EV/IV video and photos back to “Earth” through xGDS. (Voice loops between EV/IV were transmitted to the SST through an auxiliary software system and were included on the video.) The SST also created notes based on EV/IV verbal descriptions and imagery for potential sample candidate locations. To facilitate discussion on these potential sample candidate locations, Minerva had several features aimed at coalescing all the scientific observations generated during this EVA phase. First, all photos could be tagged, identifying their numbering (for example, “BB” in Fig. 11). Second, all science data could have associated notes. For instance, photos of potential sample candidate locations could have SST-created notes that identified features apparent in the imagery. Third, notes could be created independently of science data; that is, scientific observation could be verbal communications between EV and IV or simply of some interesting feature detected in the streaming video. Since all the data products in xGDS automatically had time and location tags, all the pertinent observations could be correlated, improving users' ability to better associate scientific data products.

**FIG. 12.**
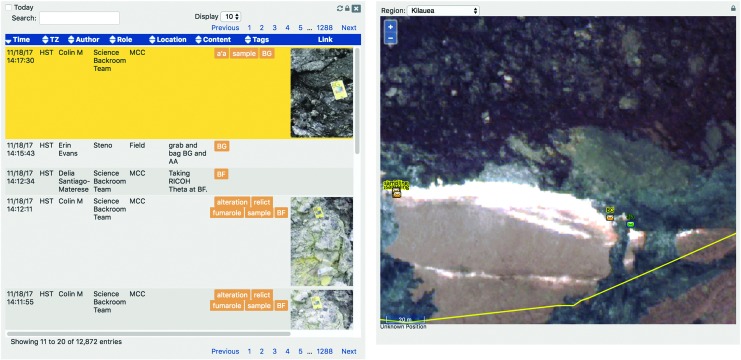
Notes with tags and geolocation in xGDS.

Notes within xGDS allowed for the multidisciplinary science team to provide their scientific observations while still being able to access and read others. As shown in Fig. 12, the biology science lead annotated the photo based on the sample's colors or wear, while a geology science lead might comment on the limited sample sizes available at that location. The capability for everyone to input notes allowed for the multiple scientific perspectives to be captured. IVs could also input notes into xGDS based on their observations. Since video, photos, and notes were all in xGDS, IVs could verify that the photos were being stored (and hence, transmitted over communication latency) to the SST, while the SST could quickly search and review the multiple, pertinent observations across the team and find the “right” photo that may lead them to decide which sample sites should be presampled.

Once the SST decided where they preferred EVs to conduct presampling (*i.e.,* collect additional instrument data from specific locations), these priorities were communicated to IV, who in turn directed the EV appropriately. There were several handheld instruments (see Sehlke *et al.*, 2019, for details about instruments) with different data outputs. Currently, only some of the data from some of the instruments were imported automatically into Minerva through xGDS. Other instrument data outputs were photographed and/or verbally read aloud by the EVs (as opposed to digitally sending data). Again, the SST notes were leveraged to annotate data and quickly share potential viability of these locations being the appropriate sampling locations. This additional information allowed the SST to reach their second major decision: preferred sample candidate locations (Fig. 10). Deciding on the sample locations required the science team to integrate the EVA's science priorities, the scientific information available at the time, and arrive at a consensus. It is important to note that both SST decisions had to be made before the start of the next EVA phase and take into account the communication latency between “Earth” and “Mars.”

Science Support Team decisions required discussions among the various scientists. They methodically determined a set of priority sampling sites, ranked in a leaderboard. The leaderboard (see also Stevens *et al.,* 2019) was a list shared in the MSC and was periodically updated by the SST with rankings and justifications as new scientific information arrived from EV and IV. When the SST arrived at a consensus, they used Playbook's Mission Log to communicate the scientific intent and justification to the IVs. The science communicator (SCICOM), a member of the SST, monitored the science team discussions as they formulated their site priorities. SCICOM's role was to communicate the leaderboard back to the IV, to inform the EVs on where to conduct presampling or sampling.

Because of the communication latency between IVs and the SST, the preferred method of communication was text-based (Abercromby *et al.,* 2013b; Chappell *et al.,* 2013, 2016; Love and Reagan, 2013; Rader *et al.,* 2013), which was supported by the Playbook Mission Log in Minerva. SCICOM entered the leaderboard (specifically the preferred set of presampling or sampling candidates) into the Mission Log and sent the message to the IV. As the rankings were updated, SCICOM would advise IVs of the changing priorities. Sampling site priorities were an essential piece of information transmitted between the science team and the crew.

**FIG. 13.**
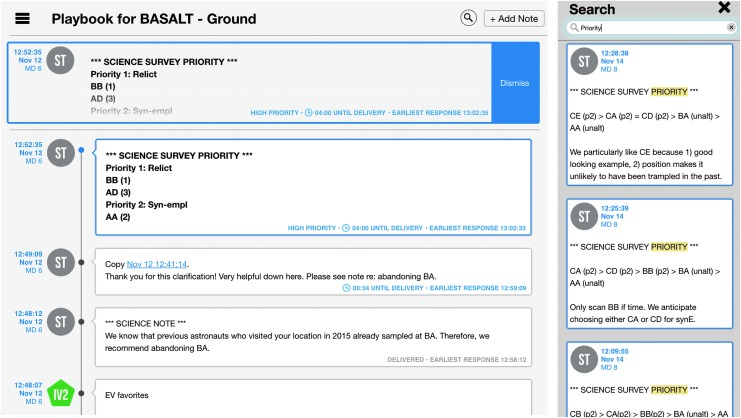
Playbook's Mission Log. Left, high-priority message on top and message delivery counters. Right, header search result within Mission Log.

When SCICOM sent a leaderboard update to IV through the Mission Log, the message would be marked as high priority. High-priority messages differ from regular Mission Log messages in that they have unique color and formatting and temporarily “float” at the top of the screen above all of the other regular messages (Fig. 13). This Mission Log feature was implemented with the intention of making important messages more salient to users. The message stood out visually and persisted longer in a designated location (*i.e.,* at the top of the screen). In turn, IVs noticed updates, communicated changes and new priorities to the EVs.

The Mission Log was used for all the communication between the SST/MSC and IVs, with the rare exception of CAPCOM or SCICOM talking to IVs through voice loop. Message content ranged from requesting angle changes to the SA camera, communicating if a particular system was unavailable, to providing additional science context or justification. With a large number of messages in the Mission Log, it was possible for the IVs to miss important messages such as the sampling priorities. To address this issue, SCICOM differentiated presampling and sampling priority messages by providing unique headers in the text, respectively: *SCIENCE SURVEY PRIORITY* and *SCIENCE SAMPLING PRIORITY* (see Kobs Nawotniak *et al.,* 2019, for more details). With the consistent use of headers, IVs leveraged Mission Log's search capability. IVs entered the unique header or subset of the header as a search term in the Mission Log, and the search results updated in real-time as new messages that matched the header arrived. This organic use of the search feature allowed IVs to have a dedicated section of the Mission Log that displayed only leaderboard update messages (Fig. 13).

**FIG. 14.**
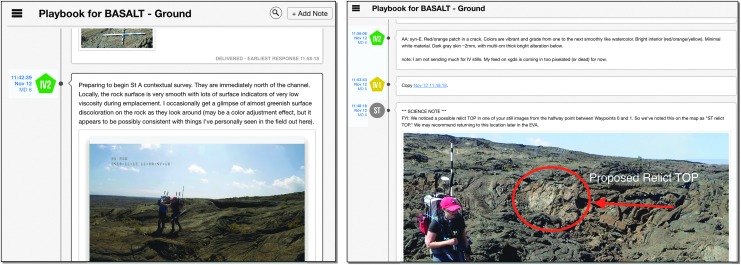
Playbook's Mission Log examples of shared images to communicate scientific information. Left, IV sending video screenshot to SST. Right, SST sending annotated photo to IV.

Occasionally, text was insufficient to communicate particular details. Images can provide a richer means to explain scientific information. Since Mission Log supported file exchange, both IVs and the SST found sharing images to be helpful, particularly during the low-bandwidth exploration condition (where the SST did not get any video from EVs). For instance, in Fig. 14, IV included a screen capture of the SA camera view for the SST. On another occasion, sharing an annotated image clarified which specific location would be an opportunistic site to explore (Fig. 14). While EVs had the capability of viewing Playbook Mission Log, they only did so if prompted by IV. There was at least one instance when the IVs asked the EVs to look on their wrist display at a shared image in Mission Log so that they could understand more precisely where the SST would like a particular sample.

The science team had a finite amount of time (*i.e.,* GAT), which varied with the communication latency, to provide their inputs to the crew. Likewise, the EVs and IVs had to keep up with the expected timeline in order to provide the science team with sufficient scientific information. The finite GAT required the science team to review and consolidate data, discuss, and arrive at a consensus decision. Minerva provided a variety of aids to facilitate decision-making, outlined below.

Aside from tracking execution and maintaining overall situation awareness of the EVA, both the science team and crew needed to understand if critical information or decisions would arrive to their counterparts in time. As mentioned, Playbook provided red lines on the timeline to provide feedback on when messages would arrive to the SST or IVs. Additionally, within the Mission Log, sent messages displayed an associated countdown timer which informed the sender how much time remained before the message arrived at its destination (*i.e.,* across the communication latency) and a time indicator that let the sender know the earliest time they should expect a response (see also Fig. 13).

As part of maintaining situation awareness, both the MSC and IVs had to understand how much time they had to impact future actions (*i.e.,* projection of future states). The IVs and EVs kept pace with the allotted time for the assigned EVA activities, trying not to significantly exceed allotted times yet not cut short the GAT, giving the SST as much time as possible to review scientific data. With regard to the SST, the scientists had a finite amount of time to provide their recommendations with regard to presampling and sampling priorities. Hence, the EVA Planner closely monitored task execution through Playbook's Timeline view, calculating how much time the science team had to make decisions and communicate priorities. Timeline management is crucial in this EVA concept of operations because if the crew finished too early or the science team was too late in making decisions, there could be a lost opportunity to affect the science collected.

**FIG. 15.**
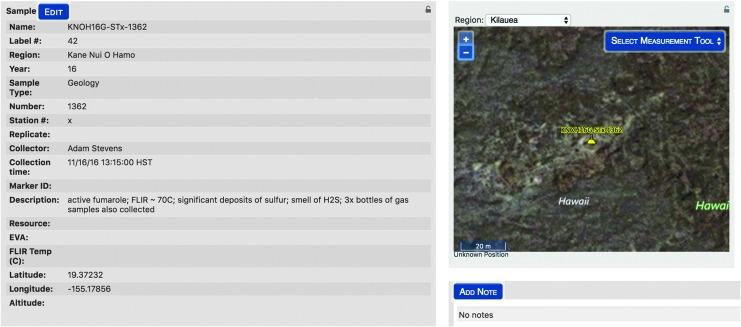
Sample metadata stored in xGDS, including time and geolocation.

Once sampling decisions were communicated to the EVs, IVs managed the identification and labeling of the many samples through Minerva. Within xGDS, IVs inputted which samples were being bagged and the label numbers of the bags. As a result, xGDS collected and managed the database of all the samples collected in the field, including time and geolocation, which were searchable after the campaign (Fig. 15).

## 5. Discussion and Future Work

Overall, Minerva successfully supported timeline, communication, and science operations management for three BASALT deployments (one in Craters of the Moon, Idaho, two in Volcanoes National Park, Hawai‘i) (see Lim *et al.,* 2019, for details). These work functions were supported under communication latency and bandwidth restrictions. Future planetary EVAs will require software systems that support all EVA work functions. We have demonstrated a subset of the critical capabilities necessary for science operations, timeline, and communication management between Earth and Mars. The consistent feedback received during the deployments was that Minerva enabled the EVA concept of operations used in BASALT, yet there are still improvements required. In this section, improvements in Minerva alongside recommendations for future EVA mission support tools are highlighted. Recommendations focus on information and functionality, as usability improvements are not the emphasis of this work.

### 5.1. Science operations management

Minerva enabled the SST to reach scientific consensus during both the planning and execution phases of EVA operations. The SST had multiple areas of expertise, ranging from organic chemistry to biology to geology. Despite their various backgrounds, scientists used the same set of shared data and information, provided by Minerva, to discuss their perspectives and agree on scientific priorities. Driven by the EVA objectives, and a SCICOM that enforced timeline deadlines, the SST provided timely sampling priorities while systematically achieving the scientific objectives set forth for the EVA. Thus, one essential recommendation for future EVA support tools is a software system that shares and automatically integrates scientific data and information in real-time in order to allow for concurrent, varied scientific interpretation.

One of Minerva's technical limitations was the capability to process and receive data from all the handheld instruments utilized by the crew. Ideally, as soon as an instrument was used, the resulting data would be transmitted to xGDS, saved within a database, and then made accessible (after the appropriate communication latency) to the scientists, just like the rest of the data sent from “Mars” (*e.g.,* still photos). Unfortunately, this was not possible for all the instruments^7^. In several cases, EVs had the additional task of reading out the instruments data and taking photos of the instrument's screen (an operational challenge given the lighting conditions). In turn, the SST took meticulous notes on what the EVs vocalized with regard to the instrument data. As a result, frustrations were expressed (*e.g.,* “what did they say?”), and time was spent on this task, which could have been spent further analyzing and discussing instrument results. This technical limitation highlights the required future functionality of integrating all scientific data into the science operations management software in order to further facilitate scientific decision-making. In particular, Mars instruments should seamlessly integrate and automatically broadcast their data.

Similarly, BASALT implemented two roles which were auxiliary to Minerva. A Stenographer role was created in order to capture everything that was verbally communicated by the EVs. The Stenographer sat near the IVs on the “Mars” side as an observer. Their responsibility was to capture within xGDS notes all the scientific observations and comments, which were then accessed by the SST during an EVA and by all scientists after the EVAs were executed. This would be analogous to Minerva having automatic voice-to-text transcription, a functionality that currently does not exist within the suite of tools. Future science operations management systems could have automatic transcription of any voice communications.

The other auxiliary role was Photo Tagging; their responsibility was to apply appropriate tags to the photos so that they could be quickly searched within xGDS. While some of the tagging required scientific knowledge and context (*e.g.,* rock is an unaltered sample), other tagging was execution related (*e.g.,* identifying marker). This particular type of tagging was not automatic, unlike the time stamps and geolocation references. Future planetary EVA software systems should provide as much pertinent tagging on scientific data products as necessary for useful scientific archiving as well as real-time integration. This includes tags that map EVA execution phases and identifying markers to enhance the geospatial and temporal context of scientific data products. Such a capability would allow for both real-time and *post hoc* synthesis between data product, providing better context for the collected samples.

As discussed, Minerva's xGDS photos and notes were essential for the SST as they captured scientific information, observations, and reference data. The SST was constantly viewing photos as well as inputting and searching notes. Conversely, IVs interfaced with this part of Minerva in different ways. For instance, IVs viewed photos in xGDS mostly to verify that the photos were successfully uploaded into the system^8^. Some xGDS notes were added to the photos by IV1, who managed operationally relevant information; however, it was observed that IV2, who managed scientific information, usually preferred handwritten scientific notes during EVA execution (ranging from 2–6 pages). These handwritten notes were used by IV2 to recall scientific details that they thought were worth writing down, often referring to these as shorthand to promote dialog with EV. For example, “semi-altered rock was B4” was recorded by IV2 and then incorporated into sample alteration discussions later in the EVA. While IV2 could have searched in xGDS to find the same information, the shorthand notes generated by hand provided pertinent information at a glance and in a format that was more conducive to promoting scientific discussion. There is still additional research to be conducted on how to make EVA software tools easy and manageable to use, particularly for IVs who experience high task loading throughout EVA execution.

### 5.2. Communication management

Minerva facilitated the communication exchange between EV, IV, and the SST via video, voice, and multimedia chat communication over one-way latencies of 5 and 15 minutes. Playbook's Mission Log was used extensively throughout each EVA. Between the two BASALT deployments, Mission Log improvements (*e.g.,* inclusion of high-priority messages and search) were received favorably by BASALT mission personnel. As previously discussed, one emergent operational behavior was the inclusion of headers for the Mission Log messages, which streamlined important messages (*e.g.,* priorities versus “simple” conversations). Predefined headers may be helpful to label messages, avoiding inadvertent misspellings, as well as more easily find them in the search. Of particular interest is the repeated request by the SST to have their own text communication “loop.” In Playbook's Mission Log, everyone shares and sees all messages, similar to posts in a Facebook or Twitter feed. Currently, users cannot send messages to specific individuals or create a separate group chat. Despite the high workload observed within the colocated science team, they requested their own “chat room” where they could exchange ideas in auxiliary group chats.

Photo annotation was determined to be beneficial, in particular when shared among both crew and SST. As previously mentioned, it was observed that the SST and IVs found it helpful for EVs to look at a picture posted in the Mission Log. These also happen to be photos that were annotated by the SST. The science team members downloaded the photos from xGDS, annotated them with some local image processing program (like Microsoft Paint or Apple OS X Preview), and then posted them in Mission Log. This emergent behavior would not have been possible if Playbook's Mission Log did not support multimedia file exchange as part of the chat interface.

As previously described, the MSC and IVs had to manage and consider different “time zones”: current time, time minus and/or time plus communication latency. The SST has the most challenging circumstances: they have to realize that the information they are receiving is delayed (by 5 or 15 minutes); they have to work and arrive at a consensus in real time, to affect actions that are more than 5 or 15 minutes in the future. The IVs mostly have to work and manage the EVA in local real time. While they received presampling and sampling priorities in a delayed manner, the SST managed their time appropriately; thus, priorities were rarely late. For further GAT analysis and how well the SST managed their time, see the work of Beaton *et al.* (2019).

In one observed occasion, IVs were confounded by having to think about the communication latency and how that affected SST communications. In this instance, the SST had sent the first set of priorities based on the scientific information they received up to that point. The IV2 wondered how much information the SST had seen before developing their initial set of priorities. At first, the IV2 attempted to reason about it based on the additional time information on the message (namely, “earliest response” time stamp on the Mission Log post that identified the priorities). However, the crew felt that time stamp was insufficient to resolve their question, and they were left wondering if the SST would provide another update. While there was sufficient information provided in Minerva to resolve this question, it was not a trivial task. IV2 had to correlate the priorities message sent time stamp (sent by the SST) against the EVA activity as well as search for the corresponding photo that would have been available to the SST before that sent time stamp. Additionally, it would not be the photo's time stamp (*i.e.,* the time when EVs took that image) but rather the time when the SST would have been able to see the photo (time stamp + transfer time + the communication latency).

Emergence of such a use case, along with others, provided the Minerva team insight on the required needs and capabilities to effectively communicate over communication latency. Future BASALT deployments will aim to improve team communication and situation awareness through Mission Log by a second chat loop exclusively for the science team, reordering messages as received, acknowledgement of message received, and automatic message posts when EVA phases are completed. Additionally, xGDS will aim to facilitate photo annotation directly through their interface, further simplifying the task of communicating through annotated photos.

Overall, future software systems that support communication management need to be more than “just a text interface.” The rate of information exchange coupled with the communication latency requires additional aids to help both Mars astronauts as well as Earth scientists communicate effectively and efficiently^9^. Key to those aids is the ability to support multimedia communication (text and images), the ability to highlight critical messages (visually or through easy-to-find headers), and inclusion of communication delay timers or indicators. Further investigation is still required to understand the effect of message ordering, natural groupings of conversations, and interrelating communications and EVA execution.

### 5.3. Timeline management

One of Minerva's strengths was that it integrated for the first time both geospatial and temporal planning and execution for EVA. The integration of the geospatial component of Minerva, provided mainly by xGDS, and the temporal component, provided by Playbook, was essential for BASALT supporting both strategic and tactical planning as well as data management. As this is the first integration of its kind, there are many lessons learned and areas of improved integration which are subsequently discussed.

One of the lessons learned was that imprecise EVA traverse modeling increased the likelihood of not being able to complete scientific objectives. Accurate time estimates for EVA traversing required taking into account how surface traversability affected crew's walking speed and the effect of simultaneous activity execution (*e.g.,* the additional time required to describe and take pictures of the traverse). Minerva was not able to take into account these factors. Additionally, the EVA timeline did not specify the time required to move between different locations within an EVA. In at least one instance, the traversability between different candidate sampling locations was particularly difficult and time-consuming. As a result, EVs fell behind schedule, and the SST had limited scientific information to decide on priorities. Understanding traversability and the impact on the EVA timeline plan is important, as this modeling could help predict if sample candidate locations were reachable and if there was sufficient time to complete the EVA's scientific objectives.

While EV's and IV's best judgment on traversability and traverse time estimates might have been sufficient in some situations, critical decisions like these in future EVAs will not be so trivial. Future software for planetary EVAs will require accurate estimates of traverses along the planned EVA path, and those estimates should be included in the EVA timeline, particularly when EVA life support consumables must be accurately incorporated into the decision-making process. There is still significant research to be done to develop rigorous human traverse models on planetary surfaces.

Minerva was ill-suited to support EVA traverse and timeline replanning (*i.e.,* modifying EVA in real-time). Once an EVA started, the planned traverse and timeline were followed. There was only one observed instance where real-time replanning was desired due to the predefined structure of the EVA timelines and overall study conditions. During one EVA, the crew quickly realized that they would not find appropriate sample candidates in that location. While they considered adding another location to explore, the crew felt uncomfortable adding another activity to the EVA despite having time to do so, resulting in the SST having fewer choices for sample priorities. It would have been possible to add and reschedule the new EVA activity within Playbook and create new traverse routes in xGDS. However, all of these changes would have been time-consuming to implement in a time-pressured environment, demanding additional responsibilities of IVs who have limited attentional resources during EVA execution.

In addition, replanning would have been challenging because IVs tracked EVA activity execution through a standalone spreadsheet that was not connected to the rest of Minerva. For instance, EVA timeline changes just prior to the start of the simulation did not propagate through Minerva where the SST and MSC would have expected to see the updates. (This also resulted in several observed instances of missed communications between the SST and crew.) One of the reasons why IVs used a standalone spreadsheet was that it performed relative time math calculations as the EVA activities were completed (a capability that did not exist in Minerva). The time calculations included tracking Phased Elapsed Time, time remaining at the activity level and EVA level, and time margin (over or behind) at the EVA level. These calculations were required for crew to keep on schedule. The SST and MSC viewed activity progress through Playbook, which was updated by either an out-of-sim person or the EVA Planner who listened to the progress on the time-delayed voice loops. An up-to-date EVA timeline (where activity progress and completion were indicated) was important for the science team as it was one of the methods used to maintain situation awareness of the state of the EVA.

In order for Minerva to support real-time replanning, BASALT would need to significantly invest in additional tool capabilities. While it was possible to create EVA routes and activities in xGDS and import them into Playbook, this capability was not used by the science or ops team. Edits, in the planning phase or in the execution phase, would not automatically propagate from one software component to another. There was no operational workflow defined to keep the planning products in sync among the entire BASALT team. Thus, from the onset of planning, traverse plans and activity plans were not tightly integrated to be modifiable during real-time execution. Integration improvements will be centered around strategically sharing key data between the software tools in an effort to improve the planning and operational workflow for EVAs.

Future BASALT deployments will attempt to improve upon the EVA timeline management capabilities used by IV into Playbook. Playbook already has the functionality of time calculations based on EVA as-planned, but it could also update them based on execution status. For instance, if an activity took longer than expected, Playbook could automatically visually reflect the new duration and shift all the subsequent activities to a later time to accommodate the as-run activity. This tactical EVA management aid would enable IVs to track the EVA progress in Playbook, which would then be automatically shared with the MSC. Additionally, the EVA activity progress could be sent to xGDS, to be displayed alongside the real-time traverse tracks. Tighter integration between Playbook, xGDS, and SEXTANT will hopefully reduce miscommunications between crew and the MSC and increase awareness of position as well as timeline state.

Overall, future EVA software will require better integrated timeline management aids for the IVs, MSC, SST, and potentially, EVs. The SST and MSC should be able to monitor EVA execution based on activity progress provided through software by IV and/or EV. For instance, when IV marks the activity completed, that information should be relayed back to Earth after the communication latency, allowing the MSC to accurately monitor EVA progress. EV should have access to simplified information regarding EVA progress, such as elapsed time or time margins on activities. Furthermore, improved timeline management aids should allow both crew and the MSC to quickly evaluate replanning options and their impact on science outcomes.

Finally, one recurring observation during EVA execution was the comment by members of the science team exclaiming “what's going on?” As previously mentioned, the SST had periods of high workload while having to monitor multiple screens and tools to manage science and track EVA progress. Some members were focused on the scientific data arriving from multiple information channels (visual and auditory). Some might be discussing between one or two people the potential implication of particular instrument data. Others might be searching the database to find supporting scientific data to justify sampling priorities. At any moment, scientists could lose awareness of timeline progress. For instance, they lost track of which sample marker was being presampled or how many samples had been collected. Maintaining situation awareness is important for all of the SST and MSC because it allows them to comprehend the current and projected state of the crew which impacts the decision-making. Information to quickly recover situation awareness includes where are the crew, which marker are they at, how many sample markers have they dropped, how much time do they have left, and how many samples still remain to be collected. Minerva had all of this information, yet it remains a challenge to provide this information in a succinct, efficient manner that permits members of the SST and MSC to maintain or quickly recover situation awareness and/or more closely track the EVA while minimizing their cognitive workload. Future EVA software systems should aim to improve users' situated understanding of EVA progress within the geospatial and temporal terms to enable more effective EVA execution and replanning.

## 6. Conclusion

Overall, the Minerva software suite allowed the BASALT EVAs to be successfully planned and executed with a distributed team contending with Mars-like communication latency and bandwidth restrictions. Though additional development is required, xGDS, SEXTANT, and Playbook performed according to the needs of the mission in their respective focus areas. xGDS provided scientists with the ability to create terrain-dependent traverse plans as well as manage scientific products, SEXTANT improved plan traverses based on available resource allocations, and Playbook provided a straightforward way for the entire team to execute and track the progress of the plan.

Using Minerva in BASALT has provided a wealth of knowledge on the future needs for software that supports planetary EVA planning and execution. Based on these two BASALT deployments, we provide the following recommendations shown in Table 7. Research still remains within each of these EVA work functions, some of which will be addressed in future BASALT deployments. Beyond these recommendations, there is still significant outstanding work to further integrate other EVA work functions, such as physiology and life support system management. Finally, BASALT focused on the concept of operation that involved a SST in a “real-time” manner, akin to current operations but with communication transmission delays. Future research should address the additional needs required for when crew is expected to explore more autonomously from the SST.

**Table 7.** Planetary EVA Software Recommendations

*A. Science Operations Management*	
A1	Integrated scientific information should be shareable in real-time in order to support concurrent, varied scientific interpretation.
A2	All scientific information from the planetary surface should automatically be integrated into software, *e.g.,* instrument data and voice transcriptions.
A3	Software should include real-time tagging of scientific data products both for EVA execution as well as post-EVA analysis.
A4	Software should facilitate searching and viewing of integrated scientific information across various parameters, like EVA phase or sample location.
*B. Communication Management*	
B1	Software should have aids for text communication under latency, such as timing counters and round-trip time calculations and sorting through high-priority communications.
B2	Multimedia communication should be supported, including text and images.
B3	Software should facilitate integrated multimedia communication between EVs, IVs, SST, and MSC.
*C. Timeline Management*	
C1	Accurate traverse estimates should be part of software for EVA traverse planning and should be integrated into EVA timeline.
C2	EVA timeline management software aids should be integrated across planning and execution as well as distributed between EVs, IVs, SST, and MSC.
C3	Temporal and spatial planning integration enable and support real-time EVA replanning.

## Footnotes

^1^ The backend software that enables Playbook to create activities and constraints is called Analog Score. Analog Score is a standalone software package that is based on the planning and scheduling tool used for International Space Station (ISS) crew and ground operations. It is capable of modeling resources, constraints, and activities which can subsequently be viewed and edited in Playbook.

^2^ In 2016, this was rendered via xGDS in Google Earth.

^3^ Meshing networks dynamically determine the best path to send data to its destination. For example, if EV1 and EV2 can see each other, but EV1's view of the main data relay station is blocked by terrain, then EV1's data will travel first through EV2's backpack and from there to the main data relay.

^5^ All procedures and documents resided in Playbook's Procedure repository.

^7^ Proprietary instrument data formats and technology did not allow for customized integration into xGDS.

^8^ Arguably, IVs could review the photos for their quality.

^9^ Note that within BASALT, voice communication is not managed by Minerva.

## Abbreviations Used

BASALT: Biologic Analog Science Associated with Lava Terrains
EV: extravehicular
EVA: extravehicular activity
GAT: ground assimilation time
IV: intravehicular
ISS: International Space Station
MSC: Mission Support Center
SA: situation awareness
SCICOM: science communicator
SEXTANT: Surface Exploration Traverse Analysis and Navigation Tool
SST: Science Support Team
xGDS: Exploration Ground Data Systems
